# Enzymatic Pretreatment of Byproducts from Soapstock Splitting and Glycerol Processing for Improvement of Biogas Production

**DOI:** 10.3390/molecules26226782

**Published:** 2021-11-10

**Authors:** Sebastian Borowski, Weronika Cieciura-Włoch

**Affiliations:** Department of Environmental Biotechnology, Faculty of Biotechnology and Food Science, Lodz University of Technology, 90-924 Lodz, Poland

**Keywords:** anaerobic digestion, biogas, soapstock, acid splitting wastewater, MONG

## Abstract

This study investigated acid splitting wastewater (ASW) and interphase (IF) from soapstock splitting, as well as matter organic non glycerol (MONG) from glycerol processing, as potential substrates for biogas production. Batch and semicontinuous thermophilic anaerobic digestion experiments were conducted, and the substrates were preliminary treated using commercial enzymes kindly delivered by Novozymes A/C. The greatest enhancement in the batch digestion efficiency was achieved when three preparations; EversaTransform, NovoShape, and Lecitase were applied in the hydrolysis stage, which resulted in the maximum methane yields of 937 NL/kg VS and 915 NL/kg VS obtained from IF and MONG, respectively. The co-digestion of 68% ASW, 16% IF, and 16% MONG (wet weight basis) performed at an organic loading rate (OLR) of 1.5 kg VS/m^3^/day provided an average methane yield of 515 NLCH_4_/kg VS_added_ and a volatile solid reduction of nearly 95%. A relatively high concentration of sulfates in the feed did not significantly affect the digestion performance but resulted in an increased hydrogen sulfide concentration in the biogas with the peak of 4000 ppm.

## 1. Introduction

Poland is the seventh largest producer of rape (*Brassica napus* L.) with 5.2% of the total world production of this plant [1]. The sow area of rapeseed and turnip rape in 2017 (as of June) was 914,000 ha, which accounted for 76% of the total area of industrial crops. This gave 2.79 million tons of rapeseeds, from which a total of 1.19 million tons of rapeseed oil was produced. Crude oil extracted from seeds contains many unwanted substances, has undesirable color and flavor, and needs to be refined before being used as an edible product [2]. The processing of crude oil consists of four main stages: degumming, alkali neutralization, bleaching, and deodorization [3,4]. During the neutralization step, fatty acids react with sodium hydroxide to produce a mixture, which is then separated through centrifugation into the light phase (degummed oil) and heavy phase (soapstock). The latter material accounts for 6% of treated crude oil and is the main byproduct of the refining process. Chemically, it consists of free fatty acids and salts (around 60% on a dry mass basis), monoglycerides, diglycerides, triglycerides, sterols, polyalcohols, and over 50% water. Moreover, it also contains phosphates, because, at an earlier stage of degumming, phosphoric acid is added to the crude oil to precipitate phospholipids [2,5]. Addition of a strong mineral acid to soapstock liberates free fatty acids (FFA) through splitting; however, this process generates highly acidic and oily wastewater [6]. An extremely low pH of 1–2 combined with high contents of sulfates and phosphates makes this wastewater difficult to treat, and the literature dealing with biological processing of this material is scarce [7].

KLM Energia is a Polish company, which produces free fatty acids from rapeseed oil soapstock and technical glycerin refined from crude glycerol. The products are then used as substrates for biodiesel production, components of cosmetics and pharmaceutics, or animal feed additives. The plant generates around 25 tons/day of wastewater from soapstock splitting, which consists of the light fraction (70–80% of wastewater volume) and the remaining heavy fraction. In the nomenclature used in this study, the light fraction refers to acid splitting wastewater (ASW), whereas the heavy fraction refers to interphase (IF). The average daily amount of MONG produced in KLM is 4–5 tons. Depending on the origin, crude glycerol has 30–80% purity, and the main impurities are alcohols, fatty acids, soaps and esters, spent catalysts, ash, and water [8,9,10]. All organic residues are referred to as MONG (matter organic non glycerol), which is also defined as 100% pure glycerol content (%)–water content (%)–ash content (%) [11]. Due to the abundance of fatty acids, alcohols, and other organic materials, MONG can be potentially attractive for biogas production. There have been many studies describing the use of crude glycerol for methane and hydrogen production [12,13]. It was also demonstrated that crude glycerol could be successfully applied for co-digestion with other organic wastes, including sewage sludge [9,14], pig manure [15], slaughterhouse waste [16,17], agri-food waste [18], and food waste [19]. However, no reports have been published on the use of MONG as a substrate for either mono-digestion or co-digestion and methane or hydrogen generation.

Hence, in this work, a new technological approach is proposed according to which heavy and light fractions of acidic wastewater (both produced in KLM Energia processing plant) were mixed with MONG and anaerobically treated with addition of enzymes to improve methane production. Batch and reactor experiments were performed to establish the optimal enzyme type and doses, as well as to evaluate the digestion stability and methane production rates. To the best of the authors’ knowledge, no similar investigations have been documented.

## 2. Results and Discussion

The characteristics of substrates and inoculum used for the experiments are shown in Table 1. All the substrates showed high organic matter content, especially MONG, with the concentration of volatile solids exceeding 950 g/kg. On the other hand, the amount of nitrogen was relatively low since the C/N ratio for all the investigated materials was 43–44, which was far from the 15–30 considered to be optimal for anaerobic digestion [20]. Acid splitting wastewater was abundant in sulfates and phosphates, the concentrations of which reached 80.2 g SO_4_^2−^/L and 4.8 g P/L. Likewise, high amounts of these undesired constituents were also determined in interphase. Sulfates and phosphates originated from the use of sulfuric acid for soapstock splitting and phosphoric acid in a degumming operation, as described in Section 1. A high concentration of chlorides in MONG should also be mentioned.

### 2.1. Batch Experiments

**Figure 1:**
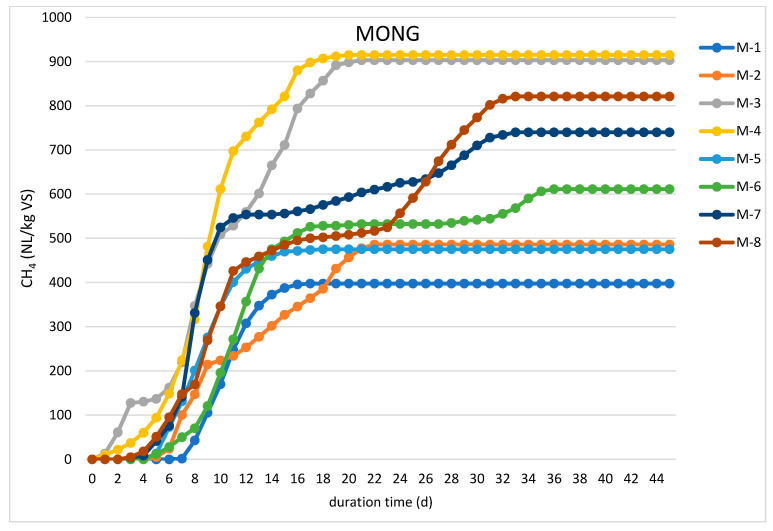
Average cumulative methane production from MONG in batch digestion experiments.

**Figure 2:**
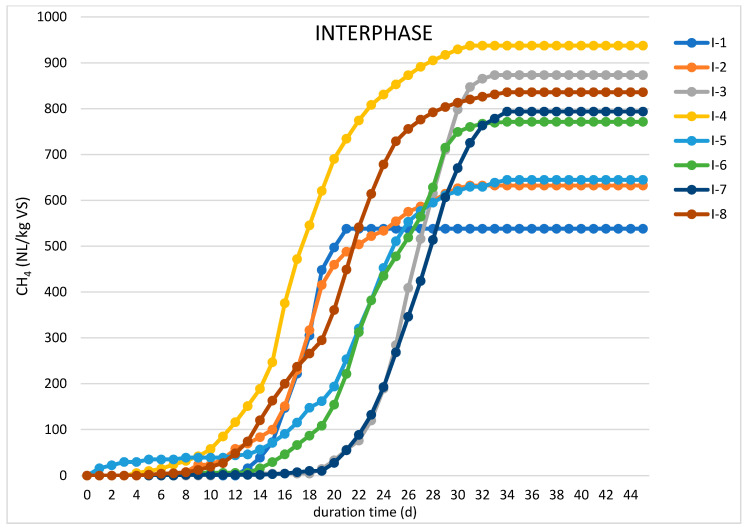
Average cumulative methane production from interphase in batch digestion experiments.

Batch tests were performed to evaluate the impact of enzymatic pretreatment on the anaerobic digestion process and methane production from individual substrates. The results of these experiments are presented in Table 2 and Figure 1 and Figure 2.

Since there was no biogas production from acid splitting wastewater due to high concentrations of sulfates (as discussed in Section 2.3), data of these runs were not included. The enzymatic hydrolysis conditions (temperature and incubation time) were established on the basis of the literature findings and manufacturer’s suggestions. Lipases are active within a wide range of temperature with a maximum activity at 50 °C for most enzymes [21,22]. It has also been reported that fatty materials are adequately hydrolyzed by lipases within 22–24 h incubation time [22,23]. As shown in Figure 1, the methane production from MONG was observed after a relatively short lag phase of 4–6 days with the maximum daily methane yields reported between days 7 and 12, which is consistent with the findings of Meng et al. [22]. Anaerobic batch digestion of MONG with no enzymatic pretreatment (M-1 run) gave nearly 400 NL/kg VS of methane, which was comparable to the yields typically obtained from crude glycerol [13]. Enzymatic pretreatment with the EversaTransform lipase dosed in an amount of 0.2 L/kg VS slightly increased this yield to 486 NL/kg VS; however, this value was still relatively low compared to methane yields from fatty materials. Surprisingly, a significant digestion improvement was achieved, when EversaTransform was applied together with NovoShape, both in doses of 0.4 L/kg VS. It is, therefore, possible that some carbohydrates present in MONG might have been decomposed by PME; however, a synergistic effect of both enzymes cannot be excluded. Moreover, the maximum methane yield of 915 NL/kg VS was reported in experiment M-4, when, in addition to the above enzymes, a small dose of Lecitase was applied. As stated by Novozymes, this preparation contains a lipase that hydrolyzes ester bonds in glycerides and liberates free fatty acids as being the main substrates for biogas production. Lecitase was also found to be efficient when applied together with NovoCor and NovoShape, and the MONG pretreatment with these enzymes resulted in a cumulative methane production of up to 821 NL/kg VS in run M-8 (Table 2, Figure 1). Furthermore, in these runs, the production of methane was observed almost since the beginning of the experiments with a relatively short lag phase.

A double curvature shape of the cumulative methane production curves can be linked to a generally slow degradation of fats as the main components of MONG. Furthermore, the shape of the curves may indicate that the digestion process occurred in two steps as a part of organic materials needed longer time for hydrolysis and degradation into methane [24,25].

The same enzyme dosing configurations were established in batch experiments with interphase, and the results of these trials are illustrated in Figure 2. In general, the results are similar to those obtained for MONG with the highest methane production of 937 NL/kg VS reported in run I-4, where interphase was enzymatically pretreated with EversaTransform, NovoShape, and Lecitase. The maximum methane yield after enzymatic pretreatment of interphase was, therefore, twofold greater than the methane production from the fresh substrate. However, in contrast to the experiments with MONG, an extended lag phase of 10 to 20 days was observed during the most batch digestion runs evaluating interphase, which might have been due to an inhibitory effect of impurities, mainly sulfates originating from sulfuric acid used for soapstock splitting.

### 2.2. Semicontinuous Experiments

**Figure 3:**
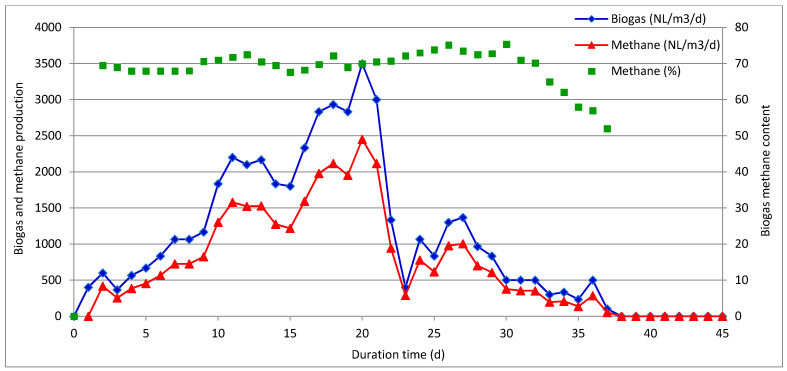
Biogas and methane production reported during the semicontinuous anaerobic co-digestion of ASW, IF, and MONG with SRT of 30 days (experiment R-1).

**Figure 4:**
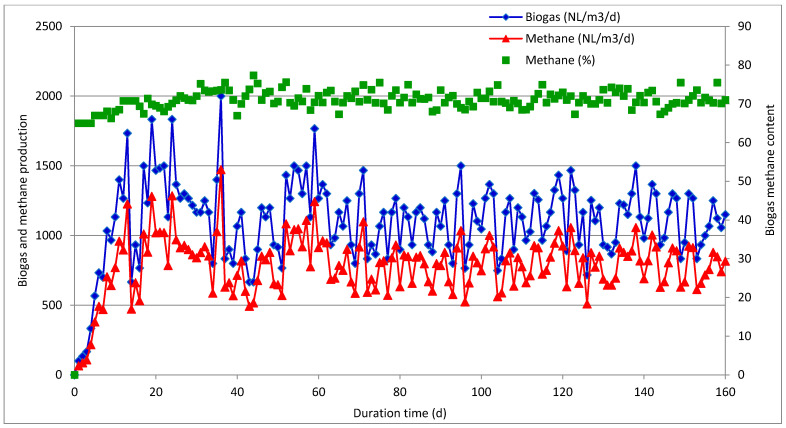
Biogas and methane production reported during the semicontinuous anaerobic co-digestion of ASW, IF, and MONG with SRT of 60 days (experiment R-2).

In the second part of this study, the individual waste materials were mixed in the proportion (wet weight basis) of 68% ASW, 16% IF, and 16% MONG, to reflect the real amounts generated by the KLM Energia company. Semicontinuous trials were performed in two reactors (herein referred to as R-1 and R-2) operated at the corresponding SRT values of 30 and 60 days. Given the highly acidic environment of the digested mixture, a sodium hydroxide solution was added to the reactors throughout the experiments to increase pH above 7. Operating parameters and performances of the semicontinuous experiments are summarized in Table 3, while the profiles of biogas and methane yields over time are plotted in Figure 3 and Figure 4.

**Figure 5:**
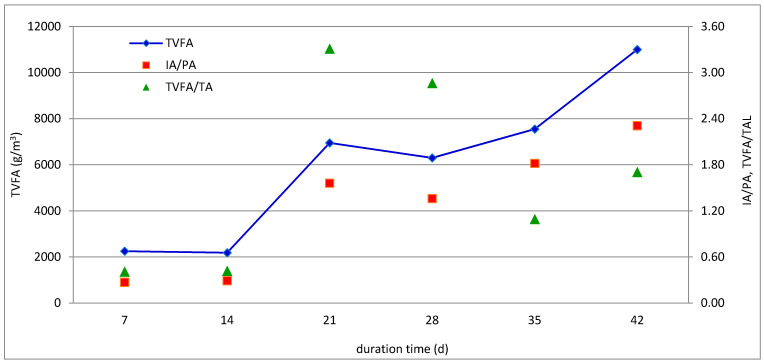
Changes in volatile fatty acids and alkalinity during the semicontinuous anaerobic co-digestion of ASW, IF, and MONG with SRT of 30 days (experiment R-1).

The reactor R-1 was operated at an SRT of 30 days and a corresponding organic loading rate (OLR) of 3 kg VS/m^3^/day. The reactor displayed unstable operation since, a gradual increase in biogas and methane yields was initially observed with peaks of 3500 NL/m^3^/day and 2500 NLCH_4_/m^3^/day, respectively, reported on day 20 of operation, which was followed by a rapid drop in the next days. These changes corresponded with variations of volatile fatty acids and alkalinity as illustrated in Figure 5. Specifically, the digester stability was evaluated using two indicators, namely, the ratio of volatile fatty acid to total alkalinity (TVFA/TA) and the ratio of intermediate to partial alkalinity (IA/PA). In order to maintain the digester stability, the TVFA/TA ratio should be maintained below 0.4–0.5, whereas IA/TA should be kept below 0.3–0.4 [15,26,27]. As shown in Figure 5, the values of both stability indicators were maintained below 0.4 for the first 2 weeks of the trial. Then, despite the highest biogas yield recorded in the third week, TVFA/TA and IA/TA increased sharply to 3.31 and 1.56, respectively, while a larger dose of NaOH was needed to maintain the pH of the digestate above 7. Finally, at the end of the trial, the IA/PA value reached 2.31, whereas the TVFA concentration exceeded 11 g/L. It seems that volatile fatty acids were mainly buffered by NaOH, as well as by ammonia, the concentration of which was the highest at the beginning of the run and gradually decreased as the experiment progressed. It is also worth mentioning that a high sulfate concentration in both feed mixture and the digestate might have also affected the anaerobic digestion process and methane production, as discussed in the next section of this paper.

**Figure 6:**
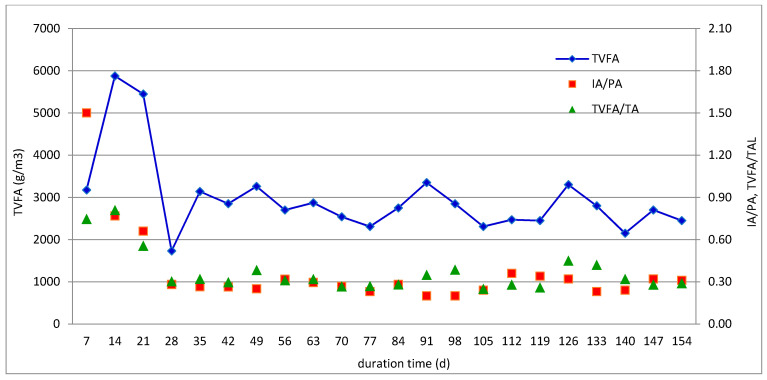
Changes in volatile fatty acids and alkalinity during the semicontinuous anaerobic co-digestion of ASW, IF, and MONG with SRT of 60 days (experiment R-2).

The semicontinuous experiment R-2 with SRT of 60 days and the corresponding OLR of 1.5 kg VS/m^3^/day exhibited an entirely different operation. Initially, the biogas production increased to 2000 NL/m^3^/day within 30 days of the run, but then stabilized at around 1100 NL/m^3^/day with the average methane percentage of 71%, as illustrated in Figure 4. The TVFA/TA and IA/TA ratios were below 0.4, whereas the average TVFA concentration did not exceed 3000 mL/L (Figure 6, Table 4). Regarding the overall R-2 reactor performance, the average methane yield was 515 NLCH_4_/kg VS_added_, whereas the volatile solid reduction reached nearly 95% (Table 3). The production of methane was lower than the corresponding values obtained in batch trials for MONG and IF, because, in semicontinuous runs, both materials were mixed with acid splitting wastewater rich in sulfates. Moreover, batch experiments usually give greater methane yields because a high inoculum addition provides nutrients, microorganisms, and buffering substances to the substrates, as well as diluted inhibitory products. Similar values were reported for the anaerobic digesters treating crude glycerol as a similar material to MONG and IF [13]. The mesophilic anaerobic co-digestion of agri-food waste and glycerol operated with a similar loading rate of 1.85 kg VS/m^3^/day gave a methane yield of 308 LCH_4_/kg VS_added_ and a high VS removal of 97% [18]. Rodríguez-Abalde et al. [17] investigated the continuous mesophilic anaerobic digestion treating a mixture composed of 35% pig slurry, 47% pasteurized slaughterhouse waste, and 18% glycerin. The methane production obtained for this mixture reached 640 LCH_4_/kg VS_added_ and was nearly threefold greater that the yield from pig manure treated alone. In contrast, Silvestre et al. [28] obtained relatively low methane yields from the mixture containing sewage sludge and crude glycerol. The reactor operated at thermophilic temperature and OLR of 1.1–1.3 kg VS/m^3^/day produced 200–390 LCH_4_/kg VS_added_, and the VS removal rate did not exceed 73%. The treatment under mesophilic conditions did not bring any improvement in the digestion performance, as only 249–325 LCH_4_/kg VS_added_ of biogas and up to 64% of VS removal were achieved despite a stable reactor operation.

### 2.3. Fate of Sulfates and Volatile Fatty Acids

**Figure 7:**
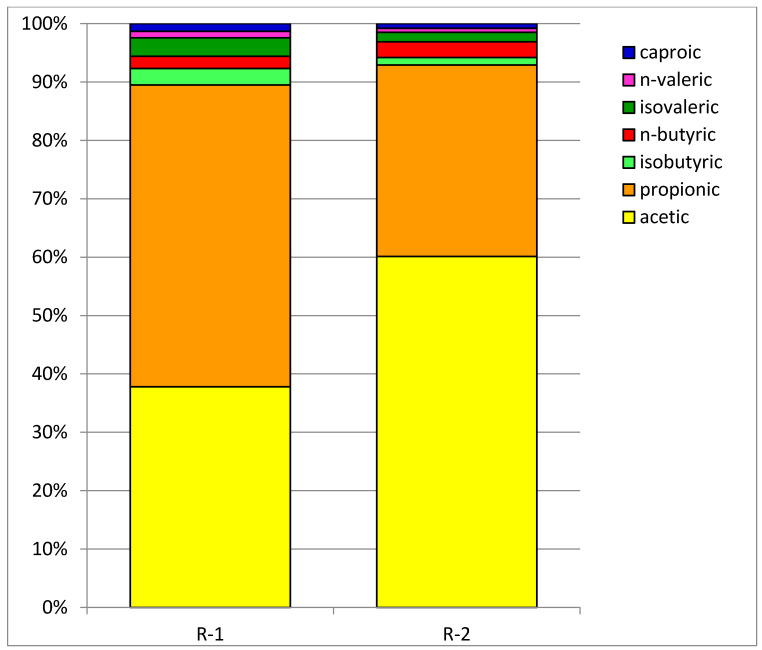
Percentages of individual volatile fatty acids in total VFA of digestates.

It is generally known that biogas production is strongly affected by the production of volatile fatty acids as the main digestion byproducts, and their increased amounts (especially propionic acid) inhibit methanogenesis. The concentrations of total volatile fatty acids along the semicontinuous runs are depicted in Figure 5 and Figure 6, whereas the profiles of individual VFAs are shown in Figure 7. As discussed earlier, during experiment R-1, a gradual accumulation of volatile fatty acids was observed, which led to the digestion process breakdown and subsequent biogas cessation. In contrast, in experiment R-2, after initial TVFA increase, the concentration of volatile fatty acids remained at a relatively constant level with an average of 2683 mg/L (Table 4). Acetic and propionic acids were the dominant VFAs accounting for around 90% TVFA in both runs, whereas the other acids were detected in much lower amounts (Figure 7), which is consistent with the findings of Rodríguez-Abalde et al. [17] and Zan and Hao [29]. However, in experiment R-1, propionic acid was the main VFA, which accounted for over 51% TVFA. At the start, the concentration of propionic acid did not exceed 1000 mg/L, but then increased to 3600 mg/L in the fourth week to finally reach 6250 mg/L. In experiment R-2, the main VFA was acetic acid which constituted approximately 60% TVFA with the mean concentration of 1610 mg/L, whereas the average content of propionic acid in that run was 880 mg/L and accounted for 32%. Accumulation of propionic acid during anaerobic digestion of crude glycerol was reported by Baba et al. [14]. The inhibitory effect of methane production was observed at OLR of 1.48 g COD/L/d when the propionic acid concentration increased to 2000–2500 mg/L. Similar findings were also reported by Fountoulakis et al. [30] and Rodríguez-Abalde et al. [17], who emphasized that the formation of propionate occurred at a much higher rate than its degradation, especially at the greater glycerol loading rates applied. On the other hand, the presence of sulfates in the digested material can lower propionic acid content because some sulfate-reducing bacteria (SRB) act as acetogens and convert propionates into acetates [29,31,32].

In our study, the average concentration of sulfates in the mixture delivered to both semicontinuously operated digesters was 60 g/L. During experiment R-1, this value did not change considerably as the average sulfate concentration in the digestate was 53 g/L (Table 4). Interestingly, only traces of sulfides were found in the digestate with an average of 7 mg/L. Moreover, the concentration of hydrogen sulfide in biogas was also relatively low. Only at the beginning of the R-1 run did the H_2_S biogas content reach 3000 ppm, later falling to less than 10 ppm after the process breakdown. It seems, therefore, that the activity of sulfate-reducing bacteria was limited in this run. In contrast, the concentration of sulfates in the R-2 experiment decreased to an average of 31.5 g/L, whereas the average content of sulfides was 1450 mg/L. In that run, the biogas initially contained as much as 4000 ppm of hydrogen sulfide, which corresponded to the highest methane yield, and then the H_2_S content decreased to approximately 650 ppm in a steady state period. A stimulatory or inhibitory effect of sulfates on methane production depends on their concentrations, as well as on the carbon-to-sulfate ratio. As reported in the literature, a visible reduction in biogas production for thermophilic anaerobic digestion is observed at C/SO_4_ and COD/SO_4_ ratios lower than 10 and 1.6, respectively [33], which is far lower than the corresponding values determined in our study for the mixture used in semicontinuous runs (14.8 and 6.6, respectively). The inhibitory effect of sulfates relies on competition between sulfate-reducing bacteria and methanogens, acetogens, and fermentative bacteria for intermediates, including acetates, propionates, butyrates, and hydrogen [29,34]. Moreover, sulfides produced by sulfate-reducing bacteria are toxic to methanogens, as well as to SRB themselves [31]. According to Parkin et al. [35], the inhibitory levels for dissolved sulfides are in the range of 100–800 mg/L, whereas they are 50–400 mg/L for undissociated H_2_S. On the other hand, temperature might play a significant role in the competition between SRB and methanogens. Colleran and Pender [36] studied the effect of temperature on the relationship between hydrogenotrophic methanogens and sulfate reducing bacteria. They reported dominance of SBP in mesophilic conditions, whereas methanogens outcompeted SRB at thermophilic temperature.

## 3. Materials and Methods

### 3.1. Materials and Pretreatment

Wastewater and MONG were collected from the KLM Energia processing plant (Smykówko, Poland). Anaerobically digested sludge was delivered from the Municipal Wastewater Treatment Plant in Zdunska Wola, Poland, and used as inoculum. The characteristics of substrates and inoculum are depicted in Table 1.

Four commercial enzymatic preparations, EversaTransform, NovoShape, NovoCor, and Lecitase, kindly donated by Novozymes A/C, (Bagsværd, Denmark) were applied during the experimental trials. Types and doses of these preparations were established on the basis of the manufacturer’s recommendations. According to the Novozyme product description, EversaTransform is a liquid lipase made from genetically modified *Aspergillus oryzae* with a declared activity of 10,000 propyl laurate units (PLU)/g. It has high activity in the transesterifciation of glycerides and in the esterification of free fatty acids (FFA). NovoShape is a preparation composed on one enzyme, pectin (methyl) esterase (PME), with an activity of 10,000 pectin esterase units (PEU)/g. The gene encoding this enzyme was derived from *Aspergillus aculeatus* and transferred into a production strain of *Aspergillus oryzae*. NovoCor is a lipase that can hydrolyze ester bonds in glycerides. It originates from the yeast *Candida Antarctica* A (CALA). The declared activity of NovoCor is 6000 lipase units (LU)/g. Lecitase Ultra is a preparation containing a protein-engineered carboxylic ester hydrolase from the fusion of lipase genes from *Thermomyces lanuginose* and phospholipase genes from *Fusarium oxysporum*. It exhibits activity toward both phospholipid and triglyceride structures. The declared activity of Lecitase is 10,000 LU/g.

### 3.2. Experiments

**Figure 8:**
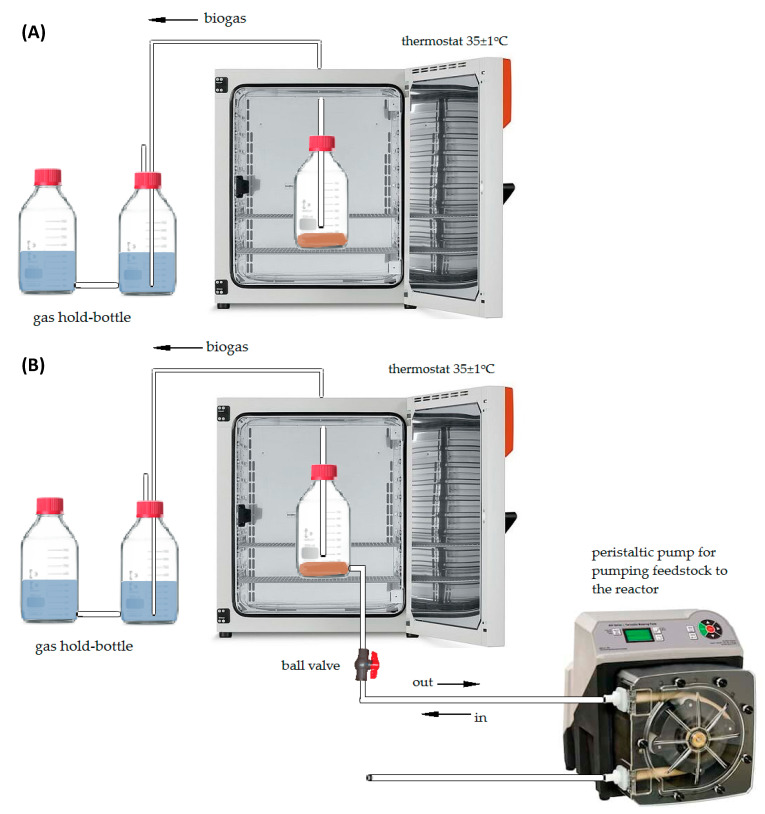
Laboratory installation for batch (**A**) and semicontinuous (**B**) experiments.

The substrates used for batch tests were preliminary treated with enzymatic preparations delivered by Novozymes (Bagsværd, Denmark). For this purpose, 50 g of each material was placed in a 200 mL flask, and then the pH value was adjusted to 7 with NaOH, followed by addition of the enzymatic preparations at doses based on the provider’s suggestions, as shown in Table 2. The flasks were incubated at 50 °C for 24 h with agitation at 150 rpm. Next, the hydrolysates were subjected to batch digestion tests performed in an installation described in the previous study [37]. Briefly, it consisted of 1 L glass bottles with a working volume of approximately 700 mL, coupled with gas hold tanks to measure daily biogas yield and to provide strict anaerobic conditions (Figure 8A). The fermentation bottles were filled with 500 g of inoculum, and then the enzymatically pretreated substrates were added at a ratio of 2:1 according to volatile solid concentration (2 g VS inoculum/1 g VS substrate) as typically applied for biomethane potential tests [38]. The bottles were placed in a thermostat and incubated at 55 °C. Each batch trial was continued to the point at which only residual or no biogas yield was measured. The individual runs were performed in triplicate, the results of which are expressed as averages.

Semicontinuous experiments (R-1 and R-2) were carried out in two identical cylindrical digesters each with total and working volumes of 3 L and 5 L, respectively (Figure 8B). They were placed in a thermostat to provide a stable temperature of 55 °C. The headspace of the digester was connected to a set of two bottles, each with a working volume of 4 L, to measure biogas production by a water displacement method. The digesters were fed once a day using a peristaltic pump. For this purpose, a certain volume of the digestate was collected, and the same amount of the feed was introduced to the reactor afterward, which provided a desired SRT. The digestates from batch tests were used as the inoculum for semicontinuous trials; hence, no special acclimation of the digesters was required. The digesters were fed with a mixture of acid splitting wastewater, interphase, and MONG in the proportion that reflected the real amounts of these materials generated by the KLM plant. Details of the digester performances are summarized in Table 3.

Three times a week, the feed mixture was preliminary treated with enzymatic preparations, whose types and doses were established in batch tests (Eversa Transform 0.4 L/kg VS, NovoShape 0.4 L/kg VS, and Lecitase 0.04 L/kg VS). The hydrolysis procedure was similar to that applied in batch tests (24 h at 50 °C with the pH adjustment to 7); however, the pretreatment process was performed using 500 g of the substrate mixture, which was then stored at 4 °C prior to use.

### 3.3. Analyses

Total and volatile solids (TS, VS), pH, and partial and total alkalinity (PA, TA) were analyzed according to standard methods [15,39]. Intermediate alkalinity (IA) was then calculated as the difference between TA and PA. Total ammonium nitrogen (TAN) and orthophosphates (PO_4_) were determined using a DR6000 spectrophotometer (HACH-Lange, Düsseldorf, Germany) with HACH-Lange tests no. 8038 and 8048, respectively. The same spectrophotometer was used to determine the concentrations of sulfates, sulfides, and chlorides according to the original HACH-Lange procedures no. 8051, 8131, and 8113, respectively. Total volatile fatty acids (TVFA) were measured spectrophotometrically with DR6000 (HACH-Lange, Düsseldorf, Germany) and HACH-Lange method no. LCK365. Individual volatile fatty acids (lactic, formic, acetic, propionic, *n*-butyric, *iso*-butyric, *n*-valeric, *iso*-valeric, and caproic) were also quantified with a high-performance liquid chromatography (HPLC) using a Finnigan Surveyor chromatograph (Thermo Scientific, San Jose, USA) coupled with an Aminex HPX 87H column (Bio-Rad, Hercules, USA) and a refractive index detector. Separation during the HPLC tests was performed using sulfuric acid (5 mmol/L) as a mobile phase, which was applied at a flow rate of 0.6 cm^3^/min. Elemental analysis (C, H, N, and P) of raw materials was performed with a Flash Elemental Analyzer, model NA2500 (CE Instruments, Wigan, UK) following the manufacturer’s procedure. The biogas yield was monitored by a water displacement method as mentioned above. The composition of the biogas was measured using a portable gas analyzer GA-21 plus (Madur Electronics, Zgierz, Poland).

The analyses of individual samples were performed in at least triplicate. The calculations of averages, standard deviations, and the analysis of variance (ANOVA) were performed in Microsoft Excel 2010. Significant differences were reported at a *p*-value lower than 0.05. A confidence level of 0.05 was selected for all statistical comparisons.

## 4. Conclusions

The enzymatic hydrolysis of fatty wastes from soapstock and glycerol processing performed at 50 °C for 24 h prior to the batch thermophilic digestion allowed a greater than twofold increase in methane yields compared to the control assays (without pretreatment). When three preparations, EversaTransform, NovoShape, and Lecitase were used for hydrolysis in the corresponding doses of 0.4, 0.4, and 0.04 L/kg VS, respectively, the methane production from IF and MONG reached 937 NL/kg VS and 915 NL/kg VS, respectively. The semicontinuous digestion process with the mixture of 68% ASW, 16% IF, and 16% MONG (wet weight basis) gave a relatively high methane yield of approximately 515 NLCH_4_/kg VS_added_ and a volatile solid reduction of as much as 95%. However, to achieve stable digestion operation, a long SRT of 60 days with the corresponding loading rate of 1.5 kg VS/m^3^/day needed to have been applied. No visible effect of the high sulfate content in the substrates on methane production was observed; however, the biogas was abundant in hydrogen sulfide due to the increased concentrations of sulfides in the digestate.

## Figures and Tables

**Table 1:** Characteristics of fresh materials and inoculum used for the experiments.

Indicator	Unit	Wastewater (ASW)	Interphase (IF)	MONG	Inoculum
pH	-	2.15 ± 0.06	6.12 ± 0.09	7.05 ± 0.11	7.28 ± 0.05
Total solids	g/kg	195.24 ± 8.92	802.40 ± 205.80	973.83 ± 13.26	26.29 ± 0.61
Volatile solids	g/kg	123.53 ± 16.77	773.73 ± 228.79	950.83 ± 34.89	21.84 ± 0.20
COD	g/kg	80.35 ± 7.25	1488.07 ± 30.85	1880.80 ± 45.20	28.19 ± 0.95
Carbon	g C/kg	121.83 ± 7.56	519.96 ± 24.72	616.43 ± 28.17	16.98 ± 0.87
Hydrogen	g H/kg	11.52 ± 0.36	54.56 ± 1.57	60.38 ± 1.92	1.52 ± 0.09
Nitrogen	g N/kg	2.83 ± 0.45	12.12 ± 1.89	13.93 ± 2.17	1.69 ± 0.28
Phosphorus	g P/kg	4.84 ± 0.56	5.33 ± 1.72	2.24 ± 0.13	0.32 ± 0.04
Ammonium nitrogen	g N/kg	0.27 ± 0.01	1.46 ± 0.02	0.19 ± 0.01	0.37 ± 0.02
Orthophosphates	g P/kg	4.78 ± 0.44	4.12 ± 0.56	1.10 ± 0.23	0.19 ± 0.03
Sulfates	g SO_4_^2−^/L	80.20 ± 8.10	28.50 ± 4.40	6.00 ± 0.50	-
Chlorides	g Cl^−^/L	0.21 ± 0.03	9.40 ± 1.25	9.35 ± 0.83	-
C/N	-	43.05	42.90	44.25	10.05
C/SO_4_^2−^	-	2.28	27.35	154.03	-
COD/SO_4_^2−^	-	1.50	78.28	469.97	-

Means ± standard deviation.

**Table 2:** Cumulative methane production obtained in batch experiments.

Experiment	Substrate	Enzyme Dose (L/kg VS)				Methane Production (NL/kg VS)
		Eversa Transform	NovoShape	NovoCor	Lecitase	
M-1	MONG	-	-	-	-	397 ± 32
M-2	MONG	0.20	-	-	-	486 ± 60
M-3	MONG	0.40	0.40	-	-	903 ± 108
M-4	MONG	0.40	0.40	-	0.04	915 ± 69
M-5	MONG	-	0.40	0.40	-	475 ± 25
M-6	MONG	-	0.40	0.80	-	611 ± 82
M-7	MONG	-	0.40	0.40	0.04	740 ± 50
M-8	MONG	-	0.80	0.80	0.08	821 ± 105
I-1	Interphase	-	-	-	-	538 ± 68
I-2	Interphase	0.20	-	-	-	632 ± 30
I-3	Interphase	0.40	0.40	-	-	873 ± 80
I-4	Interphase	0.40	0.40	-	0.04	937 ± 55
I-5	Interphase	-	0.40	0.40	-	645 ± 52
I-6	Interphase	-	0.40	0.80	-	771 ± 78
I-7	Interphase	-	0.40	0.40	0.04	794 ± 90
I-8	Interphase	-	0.80	0.80	0.08	836 ± 79

Means ± standard deviation.

**Table 3:** Operating parameters and performances of semicontinuous experiments.

Parameter	Unit	R-1	R-2
Solid retention time (SRT)	d	30	60
Organic loading rate (OLR)	kg VS/m^3^/day	3.0	1.5
Biogas production	NL/m^3^/day	1241 ± 916	1109 ± 287
	NL/kg VS_added_	405 ± 264	723 ± 188
	NL/kg VS_reduced_	451 ± 282	763 ± 235
Methane production	NLCH_4_/kg VS_added_	298 ± 195	515 ± 135
Methane biogas content	%	69.1 ± 2.6	71.0 ± 2.3
VS reduction	%	89.8 ± 6.4	94.8 ± 3.5

Means ± standard deviation.

**Table 4:** Characteristics of digestates from semicontinuous experiments.

Indicator	Unit	R-1	R-2
pH		7.15 ± 0.23	7.32 ± 0.14
Total solids	g/kg	38.17 ± 2.95	19.05 ± 1.71
Volatile solids	g/kg	18.77 ± 1.33	9.64 ± 0.68
NH_4_	mg/L	713 ± 85	1148 ± 41
PO_4_^3−^	mg/L	217 ± 32	254 ± 15
SO_4_^2−^	mg/L	53,400 ± 5643	31,500 ± 4055
S^2−^	mg/L	7 ± 3	676 ± 48
TVFA	mg/L	6057 ± 2318	2683 ± 432
PA	mg/L	1933 ± 313	6917 ± 957
TA	mg/L	4725 ± 1909	8794 ± 1199
IA/PA	-	1.4 ± 0.82	0.27 ± 0.04
VFA/TA	-	1.28 ± 1.23	0.31 ± 0.05

TVFA—total volatile fatty acids; PA—partial alkalinity; TA—total alkalinity; IA = TA − PA.

## Data Availability

Not applicable.

## Sample Availability

Samples of the compounds are not available from the authors.
